# The needs of patients with advanced, incurable cancer

**DOI:** 10.1038/sj.bjc.6605235

**Published:** 2009-08-04

**Authors:** K Rainbird, J Perkins, R Sanson-Fisher, I Rolfe, P Anseline

**Affiliations:** 1 Health Research Consultant, Dunsborough 6281, WA, Australia; 2 School of Medicine and Public Health, Faculty of Health, University of Newcastle, Newcastle 2300, NSW, Australia; 3 School of Medicine and Public Health, Faculty of Health, University of Newcastle, Newcastle 2300, NSW, Australia

**Keywords:** advanced cancer, terminal cancer patients, perceived needs

## Abstract

**Background:**

Limited research has investigated the specific needs of patients with advanced incurable cancer. The aim of this study was to describe the prevalence of perceived needs among this population.

**Methods:**

Medical specialists from two regions in New South Wales, Australia, identified patients with advanced, incurable cancer, who were estimated to have a life expectancy of <2 years and were not receiving formal palliative care. Of the 418 eligible patients, 246 (59%) consented to participate. Consenting patients completed the Needs Assessment for Advanced Cancer Patients questionnaire, which has demonstrable validity and reliability. Patients' perceived needs were assessed across the seven domains of the questionnaire: psychological, daily living, medical communication and information, symptom related, social, spiritual and financial needs.

**Results:**

Patients identified the greatest areas of need in relation to psychological and medical communication/information domains. Patients' specific needs were highest in dealing with a lack of energy and tiredness, coping with fears about the cancer spreading, and coping with frustration at not being able to do the things they used to do.

**Conclusion:**

This study indicates that patients with advanced, incurable cancer have high levels of unmet needs, especially in relation to the areas of psychological and medical communication/information needs. The data have the potential to guide the development of interventions aimed at meeting the current unmet needs of patients with advanced, incurable cancer.

Assessing and fulfilling the needs and concerns of cancer patients is an important role for health-care professionals. Research indicates that there are high levels of unmet needs among cancer patients; the most prominent relating to the provision of information and psychological support (Gustafson *et al*, 1993; Foot and Sanson-Fisher, 1995; Meredith *et al*, 1996; Sanson-Fisher *et al*, 2000; Janda *et al*, 2006; Barg *et al*, 2007). Although such research provides an indication on how to increase quality of care to cancer patients, there has been little quantitative research regarding the specific needs of patients with advanced, incurable cancer, creating uncertainty as to what areas need to be addressed when caring for this vulnerable population group.

This uncertainty is intensified by the methodological limitations found in a majority of studies with this population. Some studies failed to distinguish between ‘needs’ and ‘problems’ (MacAdam and Smith, 1987). Although patients may perceive they have a problem, they can also decide that they will endure due to circumstance and will not register a need. For example, the hair loss resulting from chemotherapy may be a problem that the patient is prepared to accept in an effort to prolong life and hence they perceive no need for help. Other research in the field has only examined one area of need (Grobe *et al*, 1982; Christ and Siegel, 1990) or one type of cancer (Murray *et al*, 2004; Aranda *et al*, 2005; Davies *et al*, 2008). Finally, other studies have relied on the retrospective reports of family members or friends (Houts *et al*, 1988), increasing the likelihood of bias created by perceptions and time.

Such deficiency in information emphasises the importance to overcome the methodological problems, so that the needs of these patients can be adequately assessed and appropriate support can be provided. This study aimed to examine the prevalence of unmet needs among a sample of patients with advanced, incurable cancer who were not receiving formal palliative care. Patients' perceived needs were assessed using a multidimensional tool with demonstrated validity and reliability.

## Materials and methods

### Participants

Forty-four medical specialists from the Sydney and Hunter regions of New South Wales in Australia recruited patients for the study. Patients were defined as eligible if their medical specialist judged that they had a life expectancy of >3 months but <2 years, and they were not under the care of the local Palliative Care Unit. Patients under the care of this unit were excluded as patients with advanced incurable cancer who are not in palliative care have been less studied. The Needs Assessment for Advanced Cancer Patients (NA-ACP) questionnaire has been validated for this population. Patients who were too physically ill, too emotionally distressed, <18 years of age or not literate in English were excluded.

### The questionnaire

The ‘Needs Assessment for Advanced Cancer Patients’ is a pen and paper questionnaire, specifically developed to assess the needs of people who have advanced, incurable cancer. The questionnaire has acceptable levels of internal consistency and test–retest reliability, as well as construct validity (Rainbird *et al*, 2005). The version of the NA-ACP used in this study comprised 132 ‘needs’ items. These items were used to calculate scores for the seven domains of possible need, psychological, medical communication/information, daily living, symptom related, social, spiritual and financial needs. Patients were asked to indicate their level of need for help for each need item on a 5-point Likert scale (1=not applicable, 2=satisfied, 3=low need, 4=moderate need and 5=high need) during the previous 4 months in an optimal health-care system. This 4-month time frame was recommended, given feedback from consumers and clinicians who indicated that such a passage of time was necessary to capture important issues for the population under study. The format of the Likert scale allows participants to indicate the following:

1. whether they have experienced a need within the specified time frame
2. whether their experienced needs have been met
3. if their needs have not been met, how much help they require (Sanson-Fisher *et al*, 2000).

### Additional questions

The Needs Assessment for Advanced Cancer Patients includes a number of demographic questions regarding the patient's age, gender, marital status, family income and level of education. Patients were also asked to identify the original site of their cancer and any treatment they had received. Two optional questions included in this section asked patients what their doctor had told them in relation to life expectancy given their disease and their own perceptions about this matter.

### Procedure

Eligible patients were given an information package by their medical specialist, who also briefly explained the study. The information package consisted of (i) a letter that outlined the objectives of the study and what participation would involve, (ii) a consent-to-contact form, to be completed if patients were interested in the study, (iii) a non-consent question sheet, which asked basic demographic questions and (iv) a reply paid envelope for the return of the relevant forms. Those patients who returned a consent-to-contact form were then sent a copy of the questionnaire to view it before deciding whether they wished to take part in the study. One week later, a trained interviewer telephoned the consenting patients and asked whether they were still willing to participate. A suitable time to visit their home was then arranged. Patients self-completed the NA-ACP, with the interviewer being present to answer any questions.

### Statistical analysis

Descriptive statistics and confidence intervals were used to analyse participant characteristics, demographics, prevalence of needs and perceptions of life expectancy. *χ*^2^ analyses were used to establish any differences between consenters, non-consenters and the 1994 NSW sample of cancer deaths (Coates and Armstrong, 1997). This report is compiled annually by the New South Wales Central Cancer Registry, and includes information about demographics, disease prevalences and characteristics. It is the most representative source of NSW cancer incidences.

## Results

### Consent rate

The medical specialists distributed 457 information packages to patients of whom 39 were excluded because: (i) they had died between the time of returning the consent-to-contact form and being contacted about taking part in the study, (*n*=21); (ii) they were now under the care of the Palliative Care Unit (*n*=9); (iii) they could not be contacted (*n*=7); or (iv) they were not literate in English (*n*=2). Of the 418 eligible patients, 246 (59%) patients gave their consent to participate. Of the 172 non-consenters, 92 (53%) completed a background question sheet, which provided basic demographic information. The obtained sample size was sufficient to ensure that need estimates were within ±6.5% of patients' true need levels with 95% confidence.

### Demographic characteristics

Patients in the study sample ranged in age from 27 to 89 years (mean=61 years, s.d. =11.9), 53% of the participants were female, 72% were married, and the most common types of cancer were breast (24%), colorectal (19%) and lung (11%).

There were no significant differences between the consenters and non-consenters in terms of gender, marital status or original cancer site. However, there was a difference on the basis of age (*χ*^2^=16.53, d.f.=5, *P*<0.01). The consenting sample included a greater proportion of patients in the age brackets of 40–49 and 50–59 years and a smaller proportion in the older age brackets of 70–79 and 80+ years.

Comparison between the study sample and the 1994 NSW sample (Coates and Armstrong, 1997) showed significant differences in relation to gender (*χ*^2^=10.75, d.f.=1, *P*<0.01), age (*χ*^2^=121.7, d.f.=5, *P*<0.01) and original cancer site (*χ*^2^=147.3, d.f.=7, *P*<0.01). The study sample was overrepresented in terms of females, those aged 40–49 and 50–59 years, and the breast, colorectal, lymph node and ovarian cancer cases. Males, those aged 70–79 and 80+ years, and those with cancer of the prostate, lung and other cancers, were underrepresented in the sample compared with the NSW sample (Table 1).

### Prevalence of needs

#### Overall needs

Analysis indicated that 95% (*n*=234) of the participants had experienced some level of need for help (that is, low, moderate or high) on one or more items. Further, 89% (*n*=219) of patients reported having a moderate or high level of need for help on at least one item.

#### Needs by domain

More than one-third of the patients reported a moderate/high level of need for help on items within the psychological or emotional domain (39–40%). Over thirty per cent had moderate/high needs on the medical communication/information domain (31–35%). The three most prevalent moderate/high needs ranged from 15 to 22% in the symptom domain and 10–30% in the financial domain. The prevalence of moderate/high need on the remaining domains ranged from 10 to 15% (financial 11–12%, spiritual 11–15% and social 10–13%). Of the non-domain-based items, the three most prevalent items related to patients' physical needs with over 40% reporting moderate/high needs in relation to dealing with lack of energy or tiredness, 28% dealing with pain and 27% dealing with feeling unwell most of the time (Table 2).

#### Needs by item

Fifty per cent of the most prevalent moderate/high need items related to the psychological or emotional domain and 45% related to medical communication/informational domain. The remaining 5% related to one of the non-domain-based items and can be related to patients' physical needs (Table 3).

The most prevalent items were: need for assistance in dealing with a lack of energy or tiredness (41%, *n*=100); coping with fears about the cancer spreading (40%, *n*=99); and coping with frustration at not being able to do the things they used to do (40%, *n*=98).

### Perceptions of life expectancy

#### The doctor's view

Two hundred and twenty-three (91%) patients responded to the question regarding what their doctor had told them about their life expectancy. Of these, 39% (*n*=88) indicated that their doctor had not discussed their life expectancy. A further 24% (*n*=53) reported that their doctor said they did not know how long they would live. Although 17% (*n*=40) responded that their doctor thought they would live for < 2years, 7% (*n*=15) indicated that their doctor thought they would live for >2 years and 2% (*n*=5) reported that their doctor did not think their cancer was life threatening. Another 2% (*n*=4) of patients had asked their doctor not to tell them how long they had to live. The remaining 8% (*n*=18) of patients gave an alternative response, such as, that they had not asked their doctor about their life expectancy; they had been told their cancer was incurable; they had been told a certain length of time some time ago; or the doctor had given a broad time frame (for example, ‘between 18 months and 5 years’).

#### Patients' own views of their life expectancy

Two hundred and twenty-six patients (92%) completed the question relating to their own perception of their life expectancy. The majority of patients (76%, *n*=172) indicated that they did not know how long they would live. A total of 5% (*n*=11) felt they would live <2 years, whereas 10% (*n*=23) believed they would live for >2 years, and 9% (*n*=20) indicated that the cancer would have no effect on the length of their life.

## Discussion

This study has identified those areas in which patients with advanced, incurable cancer have a perceived need for help or assistance. However, a number of methodological issues should be considered when reviewing the findings.

### Methodological considerations

The overall consent rate was 59%. Given the patient group, who tend to be fatigued, unwell and/or elderly, the obtained consent rate could be considered acceptable. Some quality of life studies on advanced cancer patients do not report the consent rates (Hiromoto and Dungan, 1991; Dudgeon *et al*, 1995; Gates *et al*, 1995; Vachon *et al*, 1995; Schofield *et al*, 2006). Others include their measures as part of a clinical trial or as part of hospital procedure (MacAdam and Smith, 1987; Tamburini *et al*, 1992). Consequently, rates of consent in this project are favourable (Hagerty *et al*, 2004; Aranda *et al*, 2005).

There were no significant differences in gender, marital status and original cancer site between the consenting and non-consenting patients. However, there were differences between the groups in relation to age, with the consenters including a greater proportion of patients aged 40–49 and 50–59 years and fewer patients aged 70–79 and 80+ years. This age bias is similar to that obtained in other surveys (Newell *et al*, 1998). Despite this limitation, this study addressed some of the shortcomings of the past research by focusing on patients with advanced incurable cancer and using a multidimensional self-report measure with demonstrable reliability, validity and acceptability (Rainbird *et al*, 2005).

### The needs of patients with advanced, incurable cancer

This research has shown that 95% of patients with advanced, incurable cancer have some level of perceived need for help and that they experience moderate or high needs across a variety of domains. These data suggest that the existing health-care system is not meeting the needs of these patients.

Areas of unmet need were in the psychological and the medical communication/informational domain. Up to 40% of the patients reported a moderate or high level of need in relation to these areas. Of the 20 most prevalent need items, 50% related to patients' psychological or emotional needs and 45% to medical communication or information. This result is not surprising given the nature of this patient population's prognosis. The results are similar to those in literature using the Supportive Care Needs Survey, examining, in general, the needs of cancer patients, those with brain tumours, those with melanoma and those with breast cancer (Bonevski *et al*, 1999; Sanson-Fisher *et al*, 2000; Janda *et al*, 2008; Minstrell *et al*, 2008).

Twenty-eight per cent of patients reported a need for help in relation to dealing with pain. As cancer pain can occur in conjunction with a number of other symptoms, such as depression, anxiety and experiencing fluctuating mood states, pain may underlie those psychological consequences (Grond *et al*, 1994). This result is at odds with previous reviews, in which the prevalence of cancer pain was reported as being 74% for a similar population group (Teunissen *et al*, 2007).

The most prevalent item was need for assistance in dealing with a lack of energy or tiredness, with 41% of the participants reporting a moderate to high need. This is not surprising as a lack of energy may prevent these patients from leaving the home to socialise. This lack of control over their activities has been associated with feelings of frustration, increased anxiety and depression due to tiredness or lack of energy (Langer and Rodin, 1976; Chen and Chang, 2004; Wu and Yao, 2007). Helping patients cope with this symptom should be an important aspect of care.

There are several possible reasons why patients perceive that their informational and psychological support needs are not being met. First, health-care providers may not be aware that patients have needs in these areas. Research suggests that oncologists are often unaware or do not establish a range of psychosocial, physical and informational concerns of their patients (Newell *et al*, 1998; Hagerty *et al*, 2004). Recent literature indicates that psychosocial concerns are becoming of paramount importance to many health-care providers, including oncologists (Trivedi *et al*, 2007). Second, health-care providers may have provided the required information, but the patients do not recall having received this material. Patients may avoid such information to help maintain a sense of hope (Leydon, 2000; Beadle *et al*, 2004). Another explanation may include deficits in health-care providers' training and education, system time and related financial restrictions or competing priorities. The research does provide support for this range of explanations, particularly in relation to meeting patients' informational needs (Girgis and Sanson-Fisher, 1995). Many clinicians do not feel competent at some aspects of communication skills (Girgis and Sanson-Fisher, 1995), with many reluctant to give estimations of life expectancy (Hagerty *et al*, 2005). Providing communication skills training to clinicians and other health-care providers helps increase their self-rated confidence in delivering bad news (Baile *et al*, 1999; Fallowfield and Jenkins, 2004). Clinicians may be unaware of other sources of information and help that can be provided. Allied help professionals can offer a range of services, such as information about symptom management or counselling services (Takayesu and Hutson, 2004).

An alternative testable hypothesis is that meeting the needs of this patient group is not possible, because of the traumas and demands imposed by their prognosis. To answer this question, there is a need for an intervention study in which efforts are made to meet the needs of patients by the application of best evidence and optimal psychosocial care and examine whether this results in a differential change in outcomes relating to patients' perceptions of need.

### Patients' perceptions of life expectancy

Only 5% of patients, who the clinicians thought had a life expectancy of <2 years, shared that perception. It was also found that 39% of patients reported that their doctor had not discussed their life expectancy with them. This may seem to be a concern given that a majority of patients wish to know their diagnosis no matter how bad (Bonevski and Cockburn, 1997). The findings are consistent with the literature regarding doctor–patient communication, reflecting that patients want to know more than doctors are prepared to tell (Bonevski and Cockburn, 1997). The finding is also in accord with the fact that over 30% of patients had a moderate or high need for help in terms of getting adequate information from the medical staff about the aspects of their prognosis, symptom management or information about counselling services and being able to have open discussion with their doctors.

This research illustrates the often ambiguous nature of a patient's understanding of their own prognosis and diagnosis and that the level of information needs can vary between patients. (Girgis and Sanson-Fisher, 1995; Leydon, 2000). This findings suggests the desirability for physicians to explicitly assess what each individual patient knows and would like to be told about their condition so that their information needs might be successfully met.

## Conclusions

The findings of this study indicate that patients who have advanced, incurable cancer experience high levels of need in relation to psychological/emotional and medical communication/information issues. The findings suggest the need to explore mechanisms by which the health-care system can positively respond to these justifiable needs. It also provides an example of a reliable and valid measure, the NA-ACP, which could be used by health-care providers to gain a deep understanding of their patients needs, in turn creating an opportunity to provide valuable and effective care. It is essential that strategies that attempt to reduce the needs of this vulnerable group are developed and rigorously trialed.

## Figures and Tables

**Table 1:** Sample demographic characteristics compared to non-consenters and NSW cancer deaths

**Characteristics**	**Survey sample (*n*=246) % (*n*)**	**Non-consenters (*n*=92) % (*n*)^a^**	**NSW sample (*n*=11 429) % (*n*)**
*Gender*			
Male	47 (115)	50 (46)	57 (6584)^**^
Female	53 (131)	50 (46)	43 (4925)
			
*Age (years)*			
18–39	3 (8)	2 (2)^**^	2 (250)^**^
40–49	15 (36)	6 (5)	5 (600)
50–59	25 (63)	14 (13)	12 (1349)
60–69	28 (68)	31 (28)	25 (2830)
70–79	25 (61)	37 (34)	33 (3805)
80+	4 (10)	10 (9)	23 (2595)
			
*Marital status*			
Married	72 (177)	70 (63)	—
*De Facto*	5 (12)	3 (3)	—
Widowed	14 (35)	18 (16)	—
Divorced	5 (12)	7 (6)	—
Never married	4 (10)	2 (2)	—
			
*Original cancer site* b			
Breast	24 (59)	27 (25)	8 (890)^**^
Colorectal	19 (47)	15 (14)	14 (1557)
Lung	11 (26)	22 (20)	19 (2207)
Lymph node	10 (25)	6 (5)	5 (543)
Ovary	5 (13)	4 (4)	2 (235)
Prostate	4 (9)	7 (6)	8 (906)
Mouth/throat	3 (8)	3 (3)	2 (281)
Unknown	3 (8)	1 (1)	—
Other	21 (51)	15 (14)	39 (4497)

a Some background questions were not answered by all non-consenters, thus total *n* is <92 on some characteristics.

b The original cancer site is that self-reported by patients who completed the NA-ACP and those who completed the background question sheet.

—Indicates that these data were not available.

^**^Indicates a significant overall *χ*^2^ at *P*<0.01.

Indicates cells identified through analysis of residuals as responsible for the significant overall *χ*^2^ when compared with the consenting sample.

**Table 2:** The three most prevalent moderate/high need items by domain

**Domain item**	**Percentage with a moderate/ high Need**	**95% CI (%)**
*Medical communication/information*		
Getting information about factors, which could influence the course of the cancer	35	29–42
To be fully informed about your medical test results as soon as possible	33	27–39
Receiving accurate medical judgments from the medical staff	31	26–38
		
*Psychological/emotional*		
Coping with fears about the cancer spreading	40	34–47
Coping with frustration at not being able to do the things you used to do	40	34–46
Dealing with concerns about your family's fears and worries	39	32–45
		
*Functional/daily living*		
Dealing with doing work around the house	30	24–36
Getting assistance to do your usual work	15	11–20
Getting assistance with preparing meals	10	6–14
		
*Financial*		
Dealing with concerns about your financial situation	12	8–17
Coping with organising financial matters	11	7–15
Paying the non-medical costs of your illness	11	7–15
		
*Symptoms*		
Dealing with a loss of appetite	22	17–28
Coping with difficulty eating and/or swallowing	16	12–21
Coping with lack of bladder control or bowel management	15	11–20
		
*Spiritual*		
Being able to choose the place where you want to die	15	10–20
Setting new priorities for your life	14	10–19
Dealing with spiritual issues of death and dying	11	7–15
		
*Social*		
Being able to express feelings with friends and/or family	13	9–18
Dealing with the reactions by your family and/or friends to your illness	11	7–16
Dealing with maintaining relationships with family members	10	7–15

CI=confidence interval.

**Table 3:** Twenty highest moderate or high needs for patients with advanced, incurable cancer (*n*=246)

**Rank**	**Item**	**Percentage with a moderate/ high need**	**95% CI (%)**	**Domain**
1	Dealing with a lack of energy and tiredness	41	34–47	Physical^a^
2	Coping with fears about the cancer spreading	40	34–47	Psychological/emotional
3	Coping with frustration at not being able to do the things you used to do	40	34–46	Psychological/emotional
4	Dealing with concerns about your family's fears and worries	39	32–45	Psychological/emotional
5	Getting information about factors, which could influence the course of the cancer	35	29–42	Medical communication/information
6	Dealing with fears about what is going to happen to you	35	29–41	Psychological/emotional
7	To be fully informed about your medical test results as soon as possible	33	27–39	Medical communication/information
8	Coping with fears about physical deterioration	33	27–39	Psychological/emotional
9	Dealing with feeling dependent on others	32	26–38	Psychological/emotional
10	Receiving accurate medical judgments from the medical staff	31	26–38	Medical communication/information
11	Having access to professional counselling, if you, your family or friends need it	31	26–38	Medical communication/information
12	Being able to have open discussion with your doctors	31	25–37	Medical communication/information
13	Having someone to help you to understand what is going to happen to you	31	25–37	Medical communication/information
14	Getting adequate information from the medical staff about your prognosis	31	25–37	Medical communication/information
15	Getting adequate information from the medical staff about the side effects	31	25–37	Medical communication/information
16	Wanting to share what you are going through with another person	31	25–37	Psychological/emotional
17	Coping with fears about losing your independence	31	25–37	Psychological/emotional
18	Coping with fears about pain or suffering	31	24–36	Psychological/emotional
19	Dealing with concerns about your family's ability to cope with caring for you	30	24–36	Psychological/emotional
20	Getting adequate information from the medical staff about your treatment	30	24–36	Medical communication/information

CI=confidence interval.

a This item did not load onto any specific domain, but was categorised as relating to patients' physical needs.
